# Complete Genome Sequence of a Human Metapneumovirus Isolate Collected in Brazil

**DOI:** 10.1128/genomeA.01597-17

**Published:** 2018-02-08

**Authors:** Nicholas Di Paola, Marielton dos Passos Cunha, Giuliana Stravinskas Durigon, Eitan Naaman Berezin, Edison Durigon, Danielle Bruna Leal Oliveira, Paolo Marinho de Andrade Zanotto

**Affiliations:** a Department of Microbiology, Institute of Biomedical Sciences, University of Sao Paulo, Sao Paulo, Brazil; b Department of Pediatrics, Santa Casa de Misericórdia Hospital, Sao Paulo, Brazil; c Infectious Disease Division, Children’s Institute University of Sao Paulo, HC-FMUSP, Sao Paulo, Brazil

## Abstract

Here, we present the complete genome sequence of a human metapneumovirus isolate collected from a hospitalized infant suffering from acute respiratory disease. This is the first complete genome sequence of human metapneumovirus originating from Brazil.

## GENOME ANNOUNCEMENT

Human metapneumovirus (HMPV) is a negative-sense single-stranded RNA virus and is part of the *Metapneumovirus* genus of the family *Pneumoviridae*. Originally discovered in 2001 in the Netherlands, it is a causal agent of acute respiratory infection (ARI) in primarily children (1, 2). The incidence of HMPV in ARI cases has varied by continent; reports have generally ranged from 4% to 16% (3–5). In southeastern Brazil, HMPV has been detected in 11.4% of children suffering from ARI (6).

Patients under 2 years of age were included in a prospective study of ARI surveillance. Patients with signs, symptoms, and/or a history of lower respiratory tract infections at the time of their admission were included in this study. This included any of the following clinical diagnoses: alveolar pneumonia, apnea, bronchiolitis, bronchospasm, coqueluchoide syndrome, croup, cyanosis, wheezing, and whooping cough. Several patients tested negative for all PCR diagnostic assays for common respiratory viruses. To discover the etiological pathogen and improve surveillance, patient samples were processed for next-generation sequencing.

Viral RNA was extracted from the nasopharyngeal aspirates using the QIAamp Viral RNA minikit (Qiagen, Valencia, CA, USA) and purified with DNase I and concentrated using the RNA Clean and Concentrator TM-5 kit (Zymo Research, Irvine, CA, USA). The paired-end RNA libraries were constructed and validated using the TruSeq Stranded Total RNA HT sample prep kit (Illumina, San Diego, CA, USA). Sequencing was done at the Core Facility for Scientific Research–University of São Paulo (CEFAP-USP/GENIAL) using the Illumina NextSeq platform. Each sample was barcoded individually, which allowed separation of reads for each patient. Short unpaired reads and bases and low-quality reads were removed using Trimmomatic version 0.36 (7). Paired-end reads (Phred quality score >33) were assembled *de novo* with SPAdes version 3.10 using default parameters (8).

The *de novo* assembly of isolate STA755 constructed a single contig of 13,243 nucleotides (nt) that was identified as an HMPV using BLASTn analysis. The HMPV-assembled consensus sequence had a 1,069× average depth. Using Geneious version 9.1.2, we extracted the consensus sequence of STA755 (9). When aligned with the HMPV reference genome sequence (GenBank accession number NC_004148), the sequence exceeded a 99.3% breadth of coverage with missing nucleotides at the extremes of the 5′ and 3′ untranslated regions.

A maximum-likelihood tree was estimated using FastTree version 2.1 (10) and included publicly available HMPV complete genomes from GenBank. We found that STA755 was most closely related to the HMPV genotype B2 Sabana strain sequence (GenBank accession number HM197719) that was isolated from a wild mountain gorilla in Rwanda (99% nucleotide pairwise identity) (11). The reported sequence is the first complete HMPV genome sequenced from Brazil.

### Accession number(s).

The complete genome sequence of the STA755 isolate has been submitted to GenBank under the accession number MG431250.
